# Hepatitis B and Asymptomatic Malaria Infection among Pregnant Women in a Semiurban Community of North-Central Nigeria

**DOI:** 10.1155/2021/9996885

**Published:** 2021-11-28

**Authors:** Cornelius Arome Omatola, Martin-Luther Oseni Okolo

**Affiliations:** Department of Microbiology, Kogi State University, Anyigba, P.M.B.1008, Kogi State, Nigeria

## Abstract

**Background:**

The overlap of malaria and Hepatitis B Virus (HBV) infections present a major threat to public health throughout endemic countries of tropical and sub-Saharan Africa. There is a paucity of data on the prevalence and associated factors of malaria and HBV infections among pregnant women in Ejule, a semiurban area of Nigeria. Therefore, the current study was designed to assess the seroprevalence of malaria and HBV among pregnant women attending antenatal clinics in Ejule Metropolis.

**Materials and Methods:**

In a hospital-based cross-sectional study, blood samples collected from 200 apparently healthy pregnant women at the Ilemona Clinic were screened for *Plasmodium falciparum* (*P. falciparum*) and HBsAg using histidine-rich protein 2 (HRP2) and hepatitis B surface antigen (HBsAg) rapid diagnostic tests (RDTs), respectively. Relevant sociodemographic and putative risk factor information was obtained with structured questionnaires.

**Results:**

The prevalence of the infections was 44 (22%), 5 (2.5%), and 1 (0.5%) for *P*. *falciparum* monoinfection and HBV monoinfection and coinfection, respectively. Single and concurrent infections peaked at ages 31–40 years but decreased with older ages. High *P*. *falciparum*, 31 (59.62%), and HBV 2 (3.85%) infection were observed among those without formal education. Contrary to ages, occupation, and knowledge of infection, malaria parasitemia differed significantly with lower educational qualification (*p* ≤ 0.001), being single (*p*=0.001), and inconsistent use of insecticide-treated bed nets (ITNs) (*p*=0.04, OR = 5, CI: 0.10–0.47). History of blood donation (OR = 5, *p*=0.04, CI: 1.10–32.80) and multiple sex partners (OR = 11.9, *p*=0.01, CI: 0.01–0.93) were found to be significantly associated with hepatitis B surface antigenemia rate during pregnancy. No evidence of HBV infection was observed in women with a history of HBV vaccination.

**Conclusions:**

Malaria is still highly prevalent among pregnant women due to high illiteracy and noncompliance to using ITNs. Therefore, routine screening and educating pregnant mothers are crucial in eliminating malaria in endemic settings. The low rate of hepatitis B and coinfection with malaria shows that further improvement in HBV vaccination could considerably reduce the disease burden among pregnant women.

## 1. Introduction

Malaria remains a disease of major public health concern globally. A world health report in 2020 showed malaria was responsible for about 229 million cases and 409 000 deaths worldwide [1]. Countries in sub-Saharan Africa and India with 85% of the global malaria burden bear the brunt of the disease. Globally, Nigeria has 27% of the cases and is ranked first among the six countries that accounted for more than half of all malaria cases followed by the Democratic Republic of the Congo (12%), Uganda (5%), Mozambique (4%), and Côte d'Ivoire, Angola, and Niger (3% each) [1]. In areas where malaria is endemic, pregnant women with lowered immunity are particularly at risk for malaria infection and to develop complications of the disease that results in mortality [2]. Factors such as the increased body surface and specific odor secretions during pregnancy, which may attract more mosquito bites, have been linked to a higher incidence of malaria during pregnancy than their absence [2, 3].

Hepatitis B virus (HBV) is the leading cause of preventable liver disease and death globally [4, 5]. HBV was responsible for approximately 257 million carrier cases, including active and inactive chronic forms with a global annual death rate estimated at 887 000 [4]. In Nigeria, HBV had a national prevalence of 11% in 2018, and according to the Ministry of Health, infection with the virus has become the leading silent killer due to nondetection in more than half of the nation's population who have never been tested and are not showing symptoms [6]. In 2020, the world health report showed about 20 million Nigerian population were living with HBV [7]. Viral transmission in the country is potentiated through exposure to infectious blood or blood products or by means of percutaneous exposure to sharp contamination [5].

Malaria and HBV coinfection may pose a serious public health problem among pregnant women in developing countries where both infectious diseases are endemic [8]. Despite having different transmission routes, the overlapping endemicity or geographical coincidence of both infections predispose people who reside in the endemic territory to coinfection [8]. A study by Scotto and Fazio [9] posited that since HBV is highly contagious from one infected individual to another, especially in the tropics, chances of viral transmission through the same route of transmission of *Plasmodium* vis-à-vis blood-to-blood contact, sharing needles, and blood transfusions are high. Asymptomatic malaria parasitic infection and full-blown illness with typical febrile paroxysms are the heights of the wide spectrum of clinical diseases caused by malaria parasites. People who live in malaria-endemic areas have a greater chance of developing asymptomatic malaria due to their acquired semi-immunity with submicroscopic levels of parasitemia. Coinfection and pregnancy-induced immunosuppression may likely modulate the clinical progression and severity of malaria [9]. Both malaria and HBV infections share some of their developmental stages within the hepatocytes, and such co-occurrence has been attributed to increased liver injury associated with synergistic multiplication of *P. falciparum* [10] and rapid progression of HBV infection, as well as the natural history of both diseases [11]. The mutual effect of coinfection in pregnancy aggravates clinical manifestations of malaria, which can culminate in poor pregnancy outcomes such as low birth weight, intrauterine growth restriction, maternal anemia and fetal mortality [12]. In Kogi State, reports of HBV or malaria monoinfection have been documented [13–15]. However, little or no information exists with regards to HBV and malaria coinfection in the north-central region of Nigeria, albeit in pregnant women seeking antenatal care in Ejule, Nigeria. The current study aimed to evaluate seropositivity to HBsAg and malaria parasite antigen singly and concurrently among pregnant women attending the antenatal clinic in Ejule metropolis, Kogi State, Nigeria.

## 2. Materials and Methods

### 2.1. Study Population/Design

The study was carried out at Ejule, which is situated in the eastern part of Kogi State found in the north-central region of Nigeria. Ejule town has a projected population of 24,831 people in 2020 from the 2006 population census (Center for International Earth Science Information Network, https://www.city-facts.com/ejule). Indigenes are predominantly Igala with agriculture as a major occupation. A total of 200 randomly selected apparently healthy consenting antenatal attendees aged 15–51 years were recruited at Ilemona Clinic in Ejule from September to November 2019. The hospital runs weekly services for antenatal women and is the reference hospital in the area. The number of visits to Ilemona Clinic is estimated at 180 patients/week. In this cross-sectional study, all subjects signed an informed consent form before being enrolled in the study and agreed with the publication of the findings related to the study. For participants younger than 18 years of age, their parents or guardian signed the consent form. Ethics approval for the study was obtained from the hospital management board on health issues after the due process was followed (Ethical clearance no: 058).

### 2.2. Specimen Collection and Preparation

Four (4) mL of blood was aseptically collected from each of the 200 consenting subjects by the venepuncture method into a well-labeled anticoagulant tube. The samples were transported to the microbiology department under cold conditions. The blood was spun at 3000 rpm for 8–10 minutes to separate sera from whole blood. Sera were stored at −20°C until screened for HBsAg and malaria parasite antigen. Relevant demographic variables (e.g., level of education, occupation, and marital status) and putative risk factor information (e.g., history of blood transfusion, alcoholism, mosquito net use, and multiple sexual partners) were obtained as appropriate with a closed-ended structured questionnaire with the assistance of a local language translator.

### 2.3. Laboratory Assays for HBsAg and Malaria Parasite

The commercial HBsAg test kit (ABON Biopharm Inc., Ltd., Hangzhou, China) was employed for the qualitative detection of hepatitis B surface antigen in serum. The rapid diagnostic test (RDT) kit (Standard Diagnostics, Inc., Korea) for *P. falciparum* was used to test for malaria parasites in blood samples. The RDT kit for *P. falciparum* was used since the species is the most predominant in Nigeria [1]. Analysis was carried out in the microbiology laboratory, Kogi State University, Anyigba. All test procedures and interpretation of the results were performed in accordance with the protocol of the kit's manufacturers.

### 2.4. Statistical Analysis

Statistical Packages for Social Sciences (SPSS) version 16 for windows (SPSS Inc. Chicago, IL) was used to analyze data obtained in the study. Chi-square statistics was used to compare the difference between proportions. Logistic regression analysis at a 95% confidence interval was used to determine odds ratios used to measure putative risk factors of HBV and malaria transmission. A *p* value of ≤0.05 was considered statistically significant.

## 3. Results

**Figure 1:**
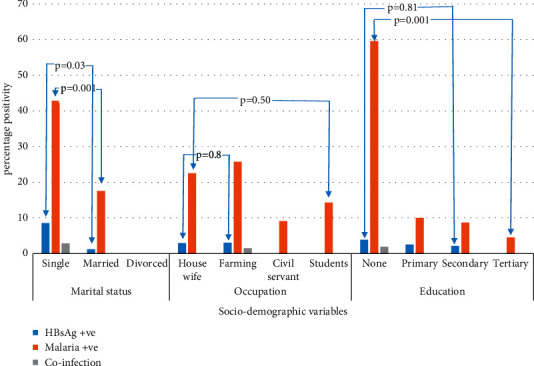
Prevalence of HBV, malaria, and coinfection in relation to socialdemographic factors.

Of the 200 study participants screened for malaria parasite and HBsAg, 44 (22%) were positive for malaria and 5 (2.5%) for HBsAg, while 1 (0.5%) sample was positive for both malaria parasite and HBsAg (Table 1). The age group 31–40 years had a higher prevalence of either HBV (3.33%), malaria (25.0%), or both infections (0.83%) than the other age groups 15–20 years with 0%, 5.88%, and 0%; 21–30 years with 2.27%, 20.45%, and 0%; 41–50 years with 0%, 21.43%, and 0%; and >50 years with 0%, 20%, and 0% for HBV, malaria, and coinfections, respectively. The presence of a malarial infection is associated with 0.9 odds (1 × 157/4 × 43 = 0.9) of being coinfected with HBV in the study. Generally, infection with HBV or malaria increased progressively from 15–20 years, peaked at ages 31–40 years, and then, decreased afterward with advancing age. Despite the increasing trend of infection with ageing, the study observed no significant difference among age groups of exposure to each infection and the prevalence rates (*p* > 0.05). In comparison with the other occupational status, women who practiced farming had a higher prevalence of malaria (25.76%) and HBV (3.03%) infection followed by housewives with HBsAg and malaria parasite positivity of 2.94% and 22.55%, respectively. A dual infection (0.5%) was observed in a woman practicing farming. A statistically significant difference was observed between the occupation of subjects and malaria/HBV coinfection (*p*=0.03). There were more malaria, 31 (59.62%), and HBV 2 (3.85%) infections among women who lacked formal education. Except HBV and HBV/malaria coinfection (*p*=0.81, 0.41), the prevalence of malaria alone differed significantly with the educational level of subjects (*p* ≤ 0.001). Generally, a decreasing trend in HBsAg and/or malaria parasitemia was observed as the level of education increases. More single women (42.86%) were infected with malaria than married (17.56%) and divorced (0.0%) women. There was a statistically significant difference between marital status and malaria infection (*p*=0.001). Similarly, more single women (8.57%) were infected with HBV than married (1.21%) and divorced (0%) women, and the difference between marital status and hepatitis B surface antigenemia rates was significant (*p*=0.03) (Figure 1). Previous exposure to donated blood was significantly linked to higher HBsAg seropositivity as women with such history had 5 times as many chances of contracting HBV infection than those without such history (OR = 5, *p*=0.04, CI: 1.10–32.80). Knowledge of HBV infection prevention tends to play a role in the study as about 3% of the seropositive subjects were those who never had prior knowledge of viral infection. Subjects with multiple sex partners had about 12 times more chances of being infected with HBV compared to those with one or two partners (OR = 11.9, *p*=0.01, CI: 0.01–0.93). The seropositivity rates to HBV were related to subjects' immunization status as 5 (2.75%) and zero prevalence rates among nonimmunized and immunized subjects, respectively, were observed in the current study (Table 2). A greater predisposition to malaria was observed among subjects who did not use mosquito net (34%) than those who use bed net consistently (10%). Seroprevalence differed significantly between consistent mosquito net users and nonusers (*p*=0.04, odds of not using mosquito net = 1/0.2 = 5, CI: 0.10–0.47) (Table 3).

## 4. Discussion

Malaria and hepatitis B virus infections are both common and life-threatening infectious diseases in developing countries including Nigeria, and the endemicity of these infections overlaps frequently due to geographical coincidence. Their high prevalence as monoinfection shows their infectious ability as coinfections. In a coinfected individual, both infections can influence each other from a clinical perspective because of some common developmental stages within the hepatocytes, which had been likened to impaired clearance of the liver stages of malaria parasites due to hepatocytes damage in hepatitis B virus infection [9, 16].

In the current study, the prevalence of malaria, hepatitis B, and coinfections was 22.0%, 2.5%, and 0.5%, respectively. The malaria prevalence in our study is comparable with the national prevalence rate of 25% [1], 22.4% in Cameroon [17], 20.4% in Ghana [18], and 28.8% earlier reported among pregnant women from other parts of the state [13]. Lower prevalence rates of 10.2%, 11.6, and 7.7% in Ethiopia, Ghana, and Nigeria, respectively, have been reported [19–21]. The plausible reason for these variations could be attributed to the marked seasonal, interannual, and spatial variability among the study areas. For instance, the current study was carried out during the rainy season with a long stretch of flooding, which could constitute suitable breeding sites for malaria vectors, causing increased vector-human interactions and malaria transmission. Contrary to our findings, relatively higher malaria parasitemia rates have been reported in other studies, in Nigeria (58%) and Ghana (42%), respectively [22, 23]. The differences in sociodemographic variables, transmission dynamics, malaria prevention measures, laboratory diagnostic tool used, adherence to intermittent preventive treatment, general health awareness, and levels of immunity in different locations are possible contributing factors to the prevalence of *P. falciparum* infections observed in the abovementioned literature.

The high rate of *P*. *falciparum* monoinfection among the less-educated women might be due to their poor lifestyle orchestrated by their inability to take adequate measures in infection control and prevention. These findings corroborate the previous report of Anabire et al. [24] in Northern Ghana. Thus, a rigorous health education program targeted at the less-enlightened women that can address the social, occupational, cultural, and economic factors associated with increased predisposition to infection could potentially control the comorbidity in most of the susceptible and underserved groups in this region and the country at large.

The rationale for the high malaria infection among women whose occupation was farming could be due to their poor awareness about infection control. Our findings are supported by the assertion of Okolo et al. [14] and Omatola et al. [15] who posited that these occupational groups, which are usually less enlightened, often overlooked the need to be properly clothed against mosquito bites during farming activities, fail to use mosquito nets consistently, and still believe in traditional rituals such as tribal marks/scarification or use of unscreened blood for transfusion that could expose them to infection.

The *P. falciparum*/HBV coinfection rate of 0.5% in the current study is lower than the prevalence rates of 4.3%, 6%, and 0.7% earlier reported in parts of Nigeria [12], Gambia [8], and Ghana [20], respectively. None of the subjects with a history of vaccination show any evidence of HBV infection, suggesting that immunity provided through HBV vaccination in the studied population could have contributed to the low HBV monoinfection rate and by implication lower comorbid state. The ability of the HBV vaccine to offer 98–100% protection against hepatitis B infection has been documented [7]. The hepatitis B surface antigenemia and its co-occurrence rate with malaria parasitemia, although low in the current study, are detrimental to the life of pregnant women and the yet unborn child. The fact that both HBV and *Plasmodium falciparum* share their life cycle in the liver suggests they could significantly influence the health and well-being of pregnant women and their fetuses in events of coinfections as has been previously reported [20]. Most babies delivered with mothers who are chronic carriers of HBV usually become chronically infected at birth if there is no prevention [25].

The high prevalence of *P*. *falciparum* monoinfection among women aged 31–40 years might be explained by their higher-risk behaviors for infection by both *Plasmodium* spp. and HBV, including sexual promiscuity, fashionable tattooing, and the lack of consistent mosquito nets used for beds than other age groups [12]. These ages coincide with the peak age for *Plasmodium* spp. and HBV coinfection suggested in a meta-analysis study of Kotepui and Kotepui [8]. The decline in mono- or coinfection with advancing age might be attributed to immunity, which is normally acquired against malaria parasites [14] or decline in the tendency for sexual promiscuity and other risky behaviors to contracting HBV infection [15]. Another possible reason is that mothers with increased age are more likely than the younger ones to have better exposure to health services and greater awareness about malaria and or hepatitis B and the ways of prevention through antenatal health talks.

We observed significant *P. falciparum* and HBV monoinfection among single women in the study area. These could be attributed to the absence of family cover, which can increase the tendency for outdoor adventures (e.g., outdoor sleeping and camping) that predisposed people to mosquito bites or unrestricted sexual activities without protection [15]. Women with multiple sex partners in the study had about 12 times odds of being monoinfected with HBV. Also, subjects with a history of blood transfusion were 5-fold more likely to be monoinfected with HBV. This finding supports the assertion of Scotto and Fazio [9] who posited that since HBV is highly contagious in the tropics, chances of viral transmission through the same route of transmission of *Plasmodium* vis-à-vis blood-to-blood contact, sharing needles, and blood transfusions are not uncommon in the endemic region. The high HBV and malaria antigenemia among women with poor knowledge of HBV or malaria infection prevention in the current study has been documented in the area [26]. Consequently, there is the need to intensify health education of the general population of Ejule on infection prevention and control strategies. Additionally, we found nonusers or inconsistent users of the insecticide-treated net with higher odds of *P. falciparum* monoinfection than consistent users, which has been reported in most studies [19, 27]. This finding might be explained by the fact that insecticide-treated nets are available to only the informed Nigerian population at a cost. Therefore, there is the need for government to make insecticide-treated mosquito nets available freely or at a subsidized rate to pregnant women and the masses to reduce both the number of malaria cases and associated sequelae.

One limitation of the study is that PCR, which is a more sensitive means of identification and confirmation of *P. falciparum* infection and occult HBV DNA, was not performed because of financial constraints. The use of less-sensitive methods suggests a higher problem on the ground and more sensitive PCR diagnostic methods should be used. Notwithstanding, the current study provides useful baseline epidemiological information regarding *P. falciparum* monoinfection and coinfection with HBV that future studies can build on in the study area. Our findings suggest the need for the initiation of routine screening of pregnant women for both hepatitis B and malaria at every antenatal care visit, as this would ensure timely management of cases and prevention of any possible adverse effects that could be associated with *P*. *falciparum*/HBV coinfection.

## 5. Conclusions

The high prevalence of malaria in the study area reaffirms its endemicity, which has been reported for most published studies in Nigeria. Low HBV monoinfection and coinfection with *P*. *falciparum* were observed. Women with the previous receipt of HBV vaccines show relative resistance to the viral infection. To considerably and rapidly reduce the burden of malaria and further decrease coinfection with hepatitis B, there is the need for government to strengthen malaria and hepatitis B prevention programs in all socioeconomic facets of the pregnant women population.

## Figures and Tables

**Table 1:** The prevalence of HBV, malaria, and coinfection in relation to ages of pregnant women in Ejule.

Age group (years)	No. tested	HBsAg +ve no. (%)	*p* value	Malaria +ve no. (%)	*p* value	Coinfection no. (%)	*p* value
15–20	17	0 (0)		1 (5.88)		0 (0)	
21–30	44	1 (2.27)		9 (20.45)		0 (0)	
31–40	120	4 (3.33)	0.086	30 (25.0)	0.51	1 (0.83)	0.96
41–50	14	0 (0)		3 (21.43)		0 (0)	
51 above	5	0 (0)		1 (20)		0 (0)	
Total	**200**	**5 (2.5)**		**44 (22.0)**		**1 (0.5)**	

**Table 2:** Logistic regression analysis of HBV infection in relation to putative risk factors.

Variable	HBsAg +ve no. (%)	OR (95% CI)	*p* value
History of blood transfusion			
Yes (*n* = 46)	3 (6.52)		
No (*n* = 154)	2 (1.30)	5.3 (1.10–32.80)	0.04
Knowledge of HBV			
Yes (*n* = 20)	0 (0)		
No (*n* = 180)	5 (2.78)	—	0.22
Alcoholism			
Yes (*n* = 42)	1 (2.38)		
No (*n* = 158)	4 (2.53)	0.9 (0.12–9.79)	0.96
Multiple sex partners			
Yes (*n* = 5)	1 (20)		
No (*n* = 195)	4 (2.05)	11.9 (0.01–0.93)	0.01
Sharing of sharp objects			
Yes (*n* = 120)	3 (2.5)		
No (*n* = 80)	2 (2.5)	1 (0.16–6.12)	1.00
Tribal mark			
Yes (*n* = 104)	2 (1.92)		
No (*n* = 96)	3 (3.13)	0.6 (0.27–10.06)	0.59
Immunized			
Yes (*n* = 18)	0 (0)		
No (*n* = 182)	5 (2.75)	—	0.48
Intravenous drug use			
Yes (*n* = 15)	0 (0)		
No (*n* = 185)	5 (2.70)	—	0.52
Surgery			
Yes (*n* = 35)	1 (2.86)		
No (*n* = 165)	4 (2.42)	1.2 (0.13–10.90)	0.88

CI = confidence interval; OR = odds ratio.

**Table 3:** Logistic regression analysis of malaria parasite infection in relation to putative risk factors.

Variables	No. tested	Malaria +ve no. (%)	OR (95% CI)	*p* value
Use of mosquito treated net				
Yes	100	10 (10)	0.2 (0.10–0.47)	0.04
No	100	34 (34)		
Knowledge of malaria				
Yes	45	9 (20)	0.9 (0.38–1.95)	0.79
No	155	35 (22.58)		

## Data Availability

The data used to support the findings of this study are included within the article.
